# Distinct genomic organization, mRNA expression and cellular localization of members of two amastin sub-families present in *Trypanosoma cruzi*

**DOI:** 10.1186/1471-2180-13-10

**Published:** 2013-01-17

**Authors:** Monica Mendes Kangussu-Marcolino, Rita Márcia Cardoso de Paiva, Patrícia Rosa Araújo, Rondon Pessoa de Mendonça-Neto, Laiane Lemos, Daniella Castanheira Bartholomeu, Renato A Mortara, Wanderson Duarte daRocha, Santuza Maria Ribeiro Teixeira

**Affiliations:** 1 Departamento de Bioquímica e Biologia Molecular, Universidade Federal do Paraná, Rua Quinze de Novembro, 1299, 80060-000, Centro Curitiba, PR, Brazil; 2 Departamento de Bioquímica e Imunologia, Av. Antônio Carlos, 6627, 31270-901, Pampulha Belo Horizonte, MG, Brazil; 3 Departamento de Parasitologia Universidade Federal de Minas Gerais, Av. Antônio Carlos, 6627, 31270-901, Pampulha Belo Horizonte, MG, Brazil; 4 Departamento de Microbiologia, Imunologia e Parasitologia, Escola Paulista de Medicina, Universidade Federal de São Paulo, Brazil, 04021-001, São Paulo, Brazil

**Keywords:** *Trypanosoma cruzi*, Amastigote, Amastin, mRNA

## Abstract

**Background:**

Amastins are surface glycoproteins (approximately 180 residues long) initially described in *Trypanosoma cruzi* as particularly abundant during the amastigote stage of this protozoan parasite. Subsequently, they have been found to be encoded by large gene families also present in the genomes of several species of *Leishmania* and in other Trypanosomatids. Although most amastin genes are organized in clusters associated with tuzin genes and are up-regulated in the intracellular stage of *T. cruzi* and *Leishmania* spp, distinct genomic organizations and mRNA expression patterns have also been reported.

**Results:**

Based on the analysis of the complete genome sequences of two *T. cruzi* strains, we identified a total of 14 copies of amastin genes in *T. cruzi* and showed that they belong to two of the four previously described amastin subfamilies. Whereas δ-amastin genes are organized in two or more clusters with alternating copies of tuzin genes, the two copies of β-amastins are linked together in a distinct chromosome. Most *T. cruzi* amastins have similar surface localization as determined by confocal microscopy and western blot analyses. Transcript levels for δ-amastins were found to be up-regulated in amastigotes from several *T. cruzi* strains, except in the G strain, which is known to have low infection capacity. In contrast, in all strains analysed, β-amastin transcripts are more abundant in epimastigotes, the stage found in the insect vector.

**Conclusions:**

Here we showed that not only the number and diversity of *T. cruzi* amastin genes is larger than what has been predicted, but also their mode of expression during the parasite life cycle is more complex. Although most *T. cruzi* amastins have a similar surface localization, only δ-amastin genes have their expression up-regulated in amastigotes. The results showing that a sub-group of this family is up-regulated in epimastigotes, suggest that, in addition of their role in intracellular amastigotes, *T. cruzi* amastins may also serve important functions during the insect stage of the parasite life cycle. Most importantly, evidence for their role as virulence factors was also unveiled from the data showing that δ-amastin expression is down regulated in a strain presenting low infection capacity.

## Background

*Trypanosoma cruzi*, the protozoan parasite that is the etiologic agent of Chagas disease [1], undergoes four developmental stages during its complex life cycle: epimastigotes and metacyclic trypomastigotes, present in the insect vector, and intracellular amastigotes and bloodstream trypomastigotes, present in the mammalian host. This parasite must rely on a broad set of genes that allow it to multiply in the insect gut, to differentiate into forms that are able to invade and multiply inside a large number of distinct mammalian cell types and to circumvent the host immune system. To meet the challenges it faces during its life cycle, complex regulatory mechanisms must control the expression of the *T. cruzi* repertoire of about 12,000 genes. Among them, there are several large gene families encoding surface proteins, which are key players directly involved in host-parasite interactions (reviewed by Epting et al. [2]).

The amastin gene family was initially reported as a group of *T. cruzi* genes encoding 174 amino acid transmembrane glycoproteins and whose mRNA are 60-fold more abundant in amastigotes than in epimastigotes or trypomastigotes [3]. The differential expression of amastin mRNAs during the *T. cruzi* life cycle has been attributed to cis-acting elements present in the 3’UTR as well as to RNA binding proteins that may recognize this sequence [4,5]. It is also known that amastin genes alternate with genes encoding a cytoplasmic protein named tuzin [6]. After the completion of the genome sequences of several Trypanosomatids it was revealed that the amastin gene family is also present in various *Leishmania* species as well as in two related insect parasites, *Leptomonas seymouri* and *Crithidia* spp [7-9]. It has also been reported that this gene family is actually much larger in the genus *Leishmania* when compared to other Trypanosomatids. Predicted topology based on sequences found in the genomes of *L. major*, *L. infantum* and *T. cruzi* indicates that all amastins have four transmembrane regions, two extracellular domains and N- and C-terminal tails facing the cytosol [8]. Moreover, comparative analyses of amastin genes belonging to six *T. cruzi* strains evidenced that sequences encoding the hydrophilic, extracellular domain, which is less conserved, have higher intragenomic variability in strains belonging to *T. cruzi* group II and hybrid strains compared to *T. cruzi* I strains [10]. Based on phylogenetic analyses of amastin orthologs from various Trypanosomatids, it has been proposed that amastins can be classified into four subfamilies, named α-, β-, γ-, and δ- amastins. Importantly, in *L. major* and *L. infantum*, in which members of all four sub-families are found, amastin genes showed differences in genomic positions and expression patterns of their mRNAs [8,9].

More than fifteen years after their discovery, the function of amastins remains unknown. Because of the predicted structure and surface localization in the intracellular stage of *T. cruzi* and *Leishmania* spp, it has been proposed that amastins may play a role in host-parasite interactions within the mammalian cell: they could be involved in transport of ions, nutrients, across the membrane, or involved with cell signaling events that trigger parasite differentiation [9]. Its preferential expression in the intracellular stage also suggest that it may constitute a relevant antigen during parasite infection, a prediction that was confirmed by studies showing that amastins peptides elicit strong immune response during Leishmanial infection [11]. Amastin antigens are considered a relevant immune biomarker of cutaneous and visceral Leishmaniasis as well as protective antigens in mice [12].

Although complete genome sequences of two strains of *T. cruzi* (CL Brener and SylvioX-10) have been reported, their assemblies were only partially achieved because of their unusually high repeat content [13,14]. Therefore, for several multi-gene families, such as the amastin gene family, their exact number of copies is not yet known. According to the current assembly [15], only four δ-amastins and two β-amastins were identified in the CL Brener genome. Herein, we used the entire data set of sequencing reads from the CL Brener [13] and Sylvio X-10 [14] genomes, to analyzed all sequences encoding amastin orthologues present in the genomes of these two *T. cruzi* strains and determine their copy number as well as their genome organization. Expression of distinct amastin genes in fusion with the green fluorescent protein, allowed us to examine the cellular localization of different members of both amastin sub-families. By determining the levels of transcripts corresponding to each sub-family in all three parasite stages of various strains we showed that, whereas the levels of δ-amastins are up-regulated in amastigotes, β-amastin transcripts are significantly increased in the epimastigote insect stage. Most importantly, evidence indicating that amastins may constitute *T. cruzi* virulence factors was suggested by the analyses showing reduced expression of δ-amastins in amastigotes from strains known to have lower infection capacity.

## Results and discussion

### The amastin gene repertoire of *Trypanosoma cruzi*

**Figure 1:**
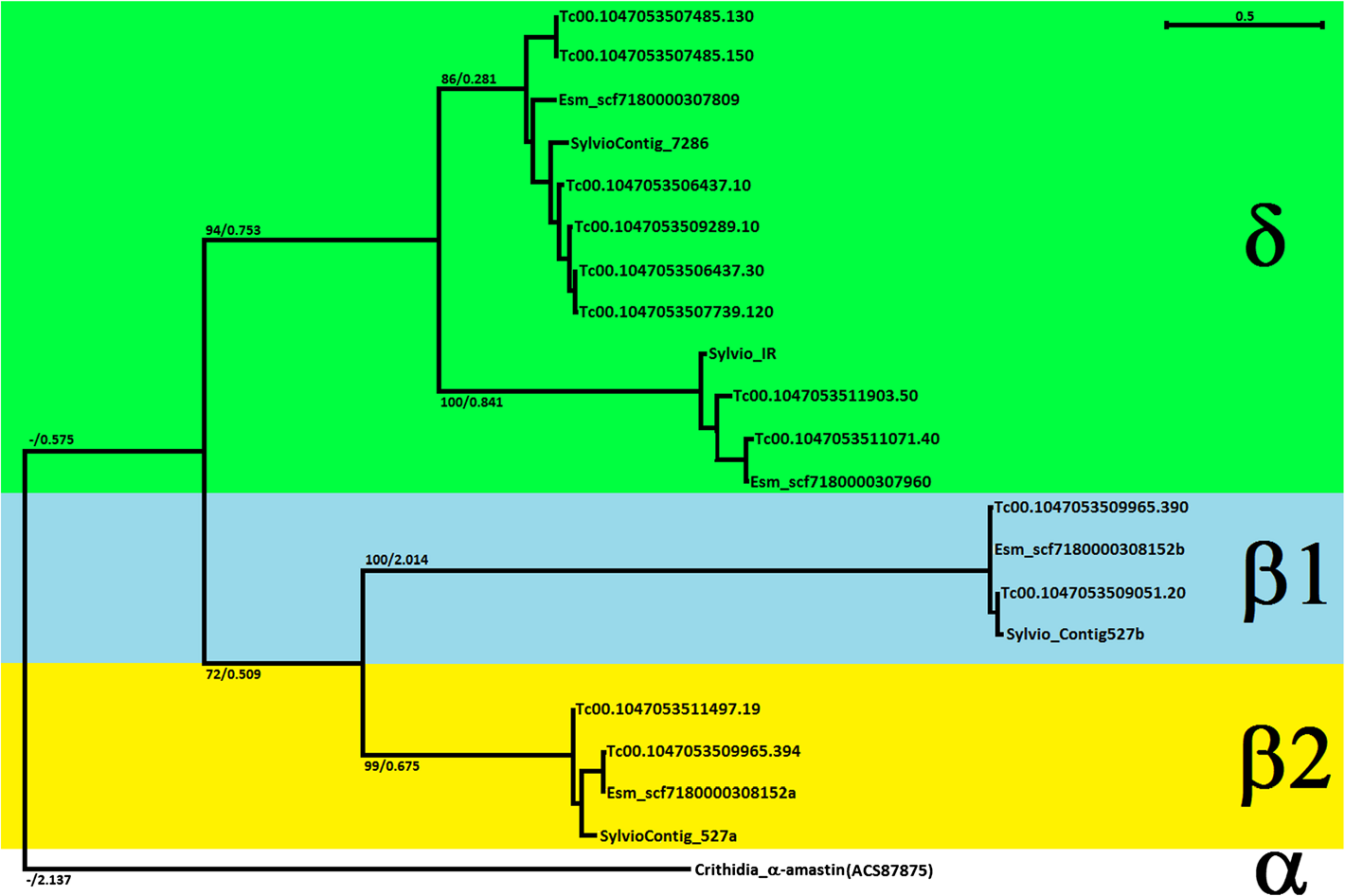
**Phylogenetic analyses of amastin sequences from different *T. cruzi* strains.** Amastin amino acid sequences from CL Brener, Esmeraldo and Sylvio X-10 strains were used to generate a tree rooted with an α-amastin sequence from *Crithidia* sp. Bootstrap values followed by branch length are shown in the major basal nodes.

In its current assembly, the *T. cruzi* (CL Brener) genome exhibits 12 putative amastin sequences. Because of its hybrid nature and the high level of divergence between alleles, this genome was assembled as two set of contigs, each corresponding to one haplotype that were denominated Esmeraldo-like and non-Esmeraldo [13]. Therefore, the 12 amastin sequences annotated in the CL Brener genome database actually correspond to 6 pairs of alleles. Based on the analyses of amastin sequences present in the genomes of different species of *Trypanosoma* and *Leishmania,* as well as in two related insect parasites (*Leptomonas seymouri* and *Crithidia* spp.), Jackson (2010) [9] proposed a classification into four amastin sub-families named α-, β-, γ- and δ-amastins. In the current annotation of the *T. cruzi* CL Brener genoma two genes that belong to the β-amastin sub-family and four genes belonging to the δ-amastin sub-family can be identified. A phylogenetic tree constructed with all 12 amastin sequences annotated in the CL Brener genome plus orthologous sequences obtained from the genome databases of the Sylvio X-10 strain and from the partial genome sequence of the Esmeraldo strain shows a clear division between β-amastin and δ-amastins sequences (Figure 1). The tree also revealed the presence, in all three genomes, of one divergent copy of δ-amastin which we identified, in the CL Brener genome, as the two alleles annotated as Tc00.1047053511071.40 and Tc00.1047053511903.50, named here as δ-Ama40 and δ-Ama50. It should be noted that, in the phylogeny proposed by Jackson (2010) [9], a group of δ-amastins that include all *T. cruzi* amastins as well as amastins from *Crithidia* spp, were grouped in a branch that was named proto-δ-amastins from which all *Leishmania* δ-amastins subsequently derived. It can also be depicted from the analyses described by Jackson (2010) [9] and the phylogenetic tree shown on Figure 1 that the two members of the β-subfamily, named β1-amastin and β2-amastin are highly divergent. Whereas among the CL Brener δ-amastins, if we exclude the two divergent alleles (δ-Ama40 and δ-Ama50), the percentage of identity ranges from 85% to 100% (See Additional file 1: Figure S1A), the average identities between the two CL Brener β-amastins range from 25% (between the two copies belonging to the Esmeraldo-like haplotype) and 18% (between the two non-Esmeraldo β-amastins). Analyses of additional sequences corresponding to δ-amastins, which were obtained from the individual reads generated during the CL Brener genome sequencing (see next paragraph), also show a sequence variability ranging from 85 to 100% when compared to the previously described δ-amastins. Besides the low homology found between β- and δ-amastins, low sequence identity is also found between δ-Ama40 and δ-Ama50 with the other members of the δ-amastin sub-family. On the other hand, sequence identities between members of the β-amastins or between members of the δ-amastin sub-families range from 83% up to 99% even when we compare amastins from two phylogenetically distant strains such as CL Brener and Sylvio X-10 (Additional file 1: Figure S1A).

In spite of the sequence divergence, an alignment of polypeptide sequences belonging to all amastin sub-families shows increased amino acid conservation within the putative hydrophobic transmembrane domains. Within the predicted extracellular domains, two highly conserved cysteine and one tryptophan residues, that are part of the 10 amino acid “amastin signature” [8], may be critical for amastin function (Additional file 1: Figure S1B). On the other hand, the more variable sequences present in the two predicted extracellular, hydrophilic domains suggest that this portion of the protein, which, in amastigotes, are in contact with the host cell cytoplasm, may interact with distinct host cell proteins.

Because the assembly of CL Brener genome does not include its complete sequence, we conducted a read-based analysis to estimate the total number of amastin genes in this strain of the parasite. It is well known that the assembly of the CL Brener genome is only accurate for non-repetitive regions, and for tandemly repeated genes, misassembles frequently occurred since most repetitive copies usually collapse into one or two copies. Therefore, we used the entire dataset of reads generated by the Tri-Tryp consortium to select reads containing sequences homologous to amastin and, based on a 13 × genome coverage [13], we estimated a total number of 14 copies of amastin genes, 2 β-amastins and 12 δ-amastins in the CL Brener genome. Similar analyses performed with sequencing reads generated by Franzen *et al.* (2011) [14] from the genome of Sylvio X-10 indicated a comparable number of copies in the genome of this *T. cruzi* I strain.

**Figure 2:**
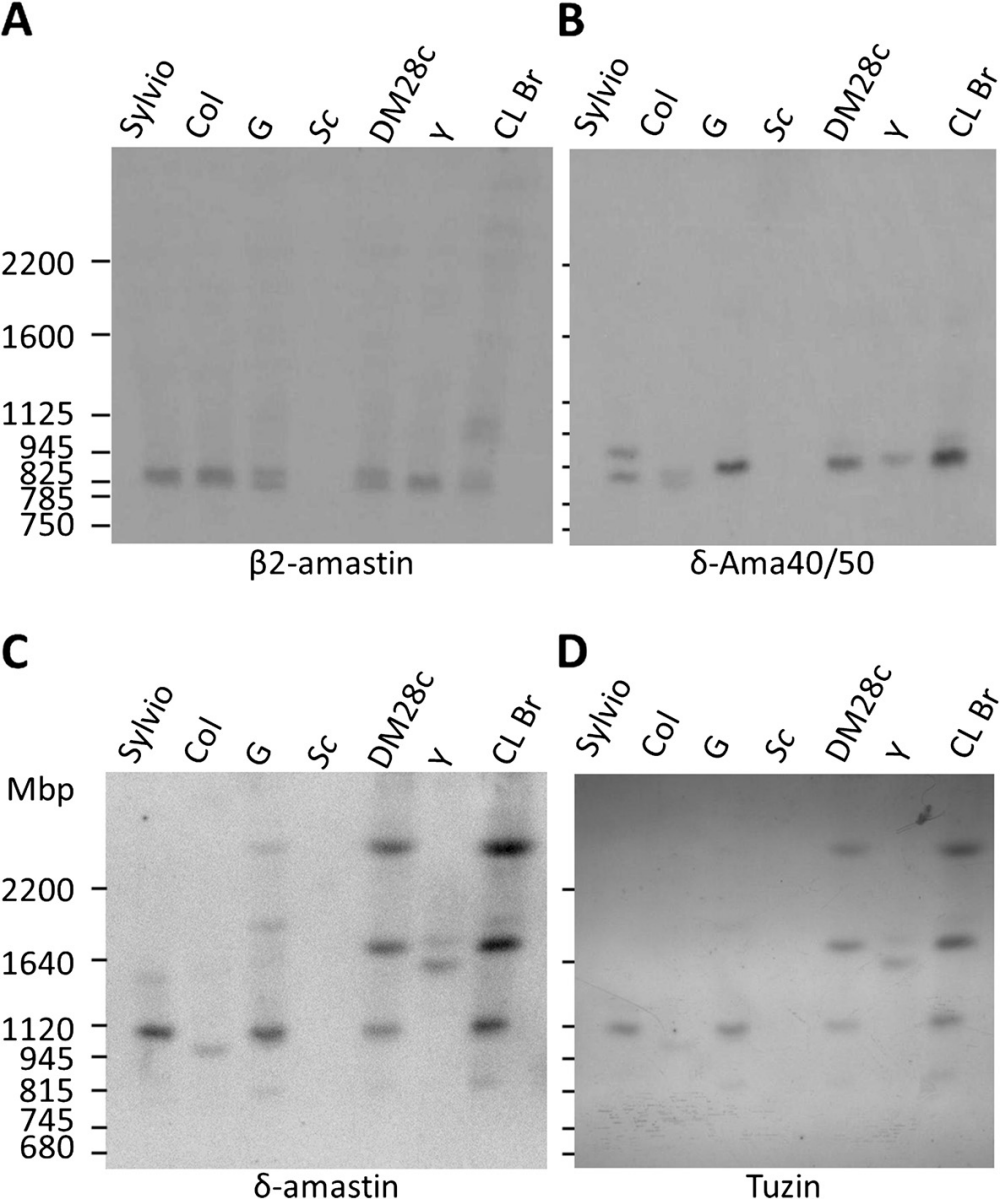
**Genomic localization of amastin genes in different *T. cruzi* strains.** Chromosomal bands from different *T. cruzi* strains, separated by Pulsed Field Gel Electrophoresis (PFGE) and transferred to membranes, were hybridized with ^32^P-labelled probes corresponding to β2-amastin **(A)**, δ-Ama40 **(B)**, δ-amastin **(C)** and tuzin genes **(D)**. *T. cruzi* strains or clones are SylvioX-10 (Sylvio), Colombiana (Col.), G and Dm28c, Y and CL Brener (CLBr). Sizes of yeast chromosomal bands (Sc) are indicated on the left.

In the current assembly of the CL Brener genome, amastin genes are shown to be organized in three *loci* on chromosomes 26, 32 and 34. Forty one pairs of homologous chromosomes (corresponding to the Esmeraldo-like and non-Esmeraldo haplotypes) have been assembled using the majority of the contigs and scaffolds generated by the Tri-Tryp consortium and inferences from synteny maps with the fully assembled *T. brucei* genome [15]. Based on the chromosome assemblies described by Weatherley et al. [15], three copies of δ-amastins are presented on chromosome 34 as a tandem array with alternating copies of tuzin genes. Interestingly, the divergent copy of δ-amastin (which has the Esmeraldo-like δ-Ama40 allele and the non-Esmeraldo allele δ-Ama50) is found as a single sequence linked to one tuzin pseudogene on chromosome 26. In a third chromosome, two copies of β-amastins are linked together without the association with tuzin genes. This gene organization is consistent with the analyses described by Jackson (2010) [9], who found tuzin genes associated only with δ-amastins. In order to confirm the proposed genomic organization in CL Brener genome and also to verify whether similar pattern of distribution of amastin genes occurs in other *T. cruzi* strains, we performed Southern blot hybridizations with chromosomal bands from CL Brener (a strain belonging to *T. cruzi* VI) as well as from G, Sylvio X-10 and Dm28c strains (all of them belonging to *T. cruzi* I) and Y strain (a *T. cruzi* II strain) separated by pulsed field gel electrophoresis. As shown in Figure 2A, the presence of two copies of β-amastins in a 900 kb chromosomal band, which is similar to the predicted size of chromosome 32 [15], has been confirmed in all *T. cruzi* strains. Using a probe specific for the δ-Ama40, we detected a chromosomal band of 800 kb, similar to the size of chromosome 26 in all strains except for the SylvioX-10, where we detected two bands of similar sizes (Figure 2B). Since significant differences in sizes of homologous chromosomal bands in *T. cruzi* have been frequently described [16], it is possible that the two bands detected in SylvioX-10 correspond to size variation of chromosome 26 from this strain. Compared to β-amastins, the pattern of distribution of δ-amastins appears to be much more complex and variable: similar to CL Brener, in Dm28c and G strains, a probe specific for δ-amastin sub-family, which does not recognizes either β-amastins or δ-Ama40/50, hybridizes with sequences present in three chromosomal bands with approximately 1.1, 1.3 and 2.3 Mb (Figure 2C). In Sylvio X-10, Colombiana and Y strains, these sequences were found in only one or two chromosomal bands. Thus, our analyses indicates that, in addition to β-amastins, which are located in chromosome 32, members of the δ-amastin sub-family are scattered among at least 3 chromosomes in this parasite strain. Whether two of these chromosomes correspond to allelic pairs that have significant differences in size, still needs to be verified. This highly heterogeneous pattern of distribution of δ-amastin sequences is also in agreement with previous analyses described by Jackson (2010) [9], which suggest that δ-amastin sequences are apparently highly mobile. Based on analyses of genomic position as well as the phylogeny of *Leishmania* amastins, it was proposed that independent movements of δ-amastins genes occurred in the genomes of different *Leishmania* species. Also consistent with these previous analyses, when blots containing chromosomal bands were probed with a sequence encoding one of the tuzin genes, a pattern of hybridization similar to the pattern obtained with the δ-amastin probes was observed (Figure 2D). Thus, for most *T. cruzi* strains, our results are consistent with the existence of more than one cluster containing linked copies of δ-amastins and tuzin genes and an additional locus with two β-amastins linked together. However, a complete description of genomic organization of amastin genes could not be attained based solely on PFGE analyses and gene copy number estimations. Further analyses based on sequencing data generated from large inserts previously mapped on specific *T. cruzi* chromosomes are warranted to solve this question.

### Distinct patterns of amastin gene expression

**Figure 3:**
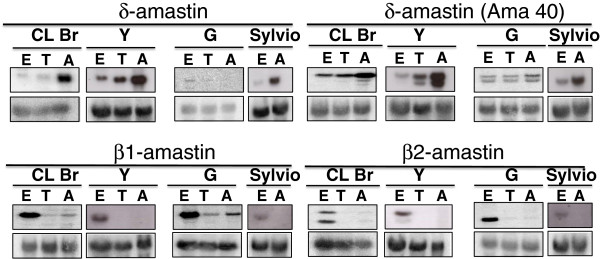
**Amastin mRNA expression during the *T. cruzi* life cycle in different parasite strains.** Total RNA was extracted from epimatigote (E), trypomastigote (T) and amastigote forms (A) from CL Brener, Y, G and Sylvio X-10. Electrophoresed RNAs (~10 μg/lane) were transferred to nylon membranes and probed with the ^32^P- labelled sequences corresponding to δ-amastin, δ-Ama40, β1- and β2-amastins (top panels). Bottom panels show hybridization of the same membranes with a fragment of the 24Sα rRNA.

Because analyses of amastin gene expression have been limited to members of the δ sub-family and these studies have not been conducted with different strains of the parasite, we decided to evaluate by northern blotting the expression profiles of members of the δ- and β-amastin sub-families. We also decided to compare the expression levels of different amastin genes in parasite strains representative of *T. cruzi* I (Sylvio X-10 and G), *T. cruzi* II (Y) and in CL Brener (a *T. cruzi* VI strain). As shown in Figure 3, the levels of amastin transcripts derived from δ- and β- sub-families are differentially modulated throughout the *T. cruzi* life cycle. Most importantly, clear differences in expression levels were found when different *T. cruzi* strains are compared: whereas in CL Brener , Y and Sylvio X-10 strains, transcripts of δ-amastins are up-regulated in amastigotes, as previously described in the initial characterization of amastins performed with the Tulahuen strain (also a *T. cruzi* VI strains) [6], the same was not observed with the G strain. Even though it presents a more divergent sequence and is transcribed from a different locus in the genome, the expression of δ-Ama40, similar to other δ-amastins, is also up-regulated in amastigotes in all strains analysed except in the G strain. In contrast, in all parasite strains, the expression of β1- and β2-amastin transcripts is up-regulated in epimastigotes. Similar to β2-amastin from CL Brener, two distinct δ-Ama40 transcripts with different sizes were detected in Y and G strains. It can be speculated that transcripts showing different sizes derived from δ-Ama40 and β2-amastin genes may result from alternative mRNA processing events. Recent reports on RNA-seq analyses indicated that alternative trans-splicing and poly-adenylation as a means of regulating gene expression and creating protein diversity frequently occur in *T. brucei*[17]. Current analyses of RNA-seq data will help elucidating mechanism responsible for the size variations observed for this sub-set of β- and δ-amastins. Moreover, the striking difference in the expression of δ-amastins observed in the G strain is also currently being investigated. Because G strain has been largely characterized as a low virulence strain [18], we speculated that members of the δ-amastins sub-family may constitute virulence factors that contributed to the infection capacity and parasite survival in the mammalian host. This hypothesis has been recently verified by experiments in which we over-expressed one δ-amastin gene in the G strain and showed that the transfected parasites have accelerated amastigote differentiation into trypomastigotes in *in vitro* infections as well as parasite dissemination in tissues after infection in mice [19]. It is also noteworthy that both β-amastins exhibited increased levels in epimastigotes of all strains analysed, indicating that this amastin isoform may be involved with parasite adaptation to the insect vector. These results are consistent with previous reports describing microarray and qRT-PCR analyses of the steady-state *T. cruzi* transcriptome, in which higher levels of β-amastins were detected in epimastigotes compared to amastigotes and trypomastigote forms [20]. Similar findings were also described for one *Leishmania infantum* amastin gene (LinJ34.0730), whose transcript was detected in higher levels in promastigotes after five days in contrast to all other amastin genes that showed higher expression levels in amastigotes [8]. The generation of knock-out parasites with the β-amastin locus deleted and pull-down assays to investigate protein interactions between the distinct *T. cruzi* amastins and host cell proteins will help elucidate the function of these proteins.

Also, to investigate the mechanisms controlling the expression of the different sub-classes of amastins, sequence alignment of the 3’UTR sequences from β- and δ-amastins were done. Previous work has identified regulatory elements in the 3’ UTR of δ-amastins as well as in other *T. cruzi* genes controlling mRNA stability [4-6,21,22] and mRNA translation [23]. Since we observed that the two groups of amastin genes have highly divergent sequences in their 3’UTR (not shown), we are preparing luciferase reporter constructs to identify regulatory elements that might be present in the β-amastin transcripts as well as to identify the factors responsible for the differences observed in the amastin gene expression in distinct *T. cruzi* strains.

### Amastin cellular localization

**Figure 4:**
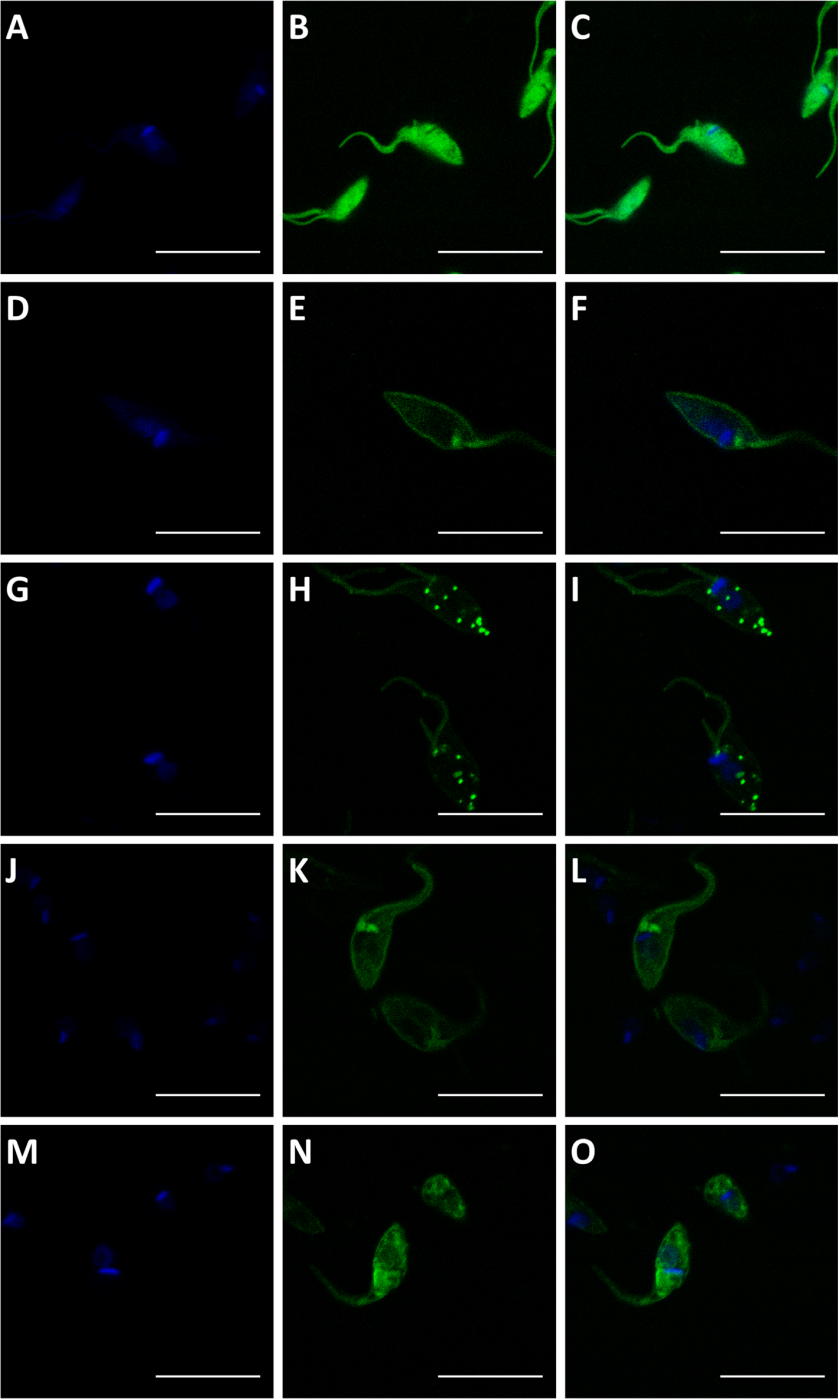
**Subcellular localization of distinct amastins in fusion with GFP.** Images from stable transfected epimastigotes of the CL Brener or G strains obtained by confocal microscopy using 1000x magnification and 2.2 digital zoom. In panels **(A-C)**, parasites transfected with a vector containing only GFP; **(D-F)**, parasites transfected with δ-amastinGFP; **(G-I)**, parasites transfected with δ-Ama40GFP; **(J-L)**, parasites transfected β1-amastinGFP; **(M-O)**, parasites transfected with β2-amastinGFP. DAPI staining are shown in panels **(A, D, G, J and M)**; GFP fluorescence in panels **(B, E, H, K and N)** and merged images in panels **(C, F, I, L and O)**. (Bar = 10 μm).

**Figure 5:**
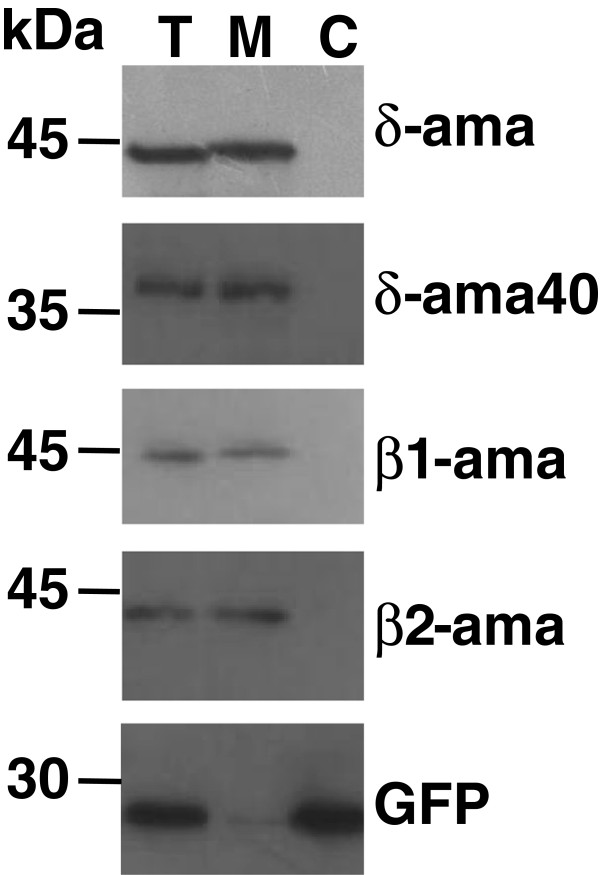
**Distribution of amastin proteins in the parasite membrane fractions.** Immunoblot of total (T), membrane (M) and cytoplasmic (C) fractions of epimastigotes expressing δ-Ama, δ-Ama40, β1- and β2-amastins in fusion with GFP. All membranes were incubated with α-GFP antibodies.

In our initial studies describing a member of the δ-amastin sub-family, we showed that this glycoprotein localizes in the plasma membrane of intracellular amastigotes [3]. Here we examine the cellular localizations of other members of the amastin family by transfecting epimastigotes of the CL Brener strain with the pTREXnGFP vector [24] containing sequences of two δ-amastins as well as β1- and β2-amastins in fusion with GFP. Using GFP fusion protein we were able to examine the cellular localization of each individual member of the family. Also, since several attempts of expressing the recombinant form of the full length proteins have been largely unsuccessful, it was not possible to generate specific antibodies that could be used to detect unambiguously each member of the distinct amastin sub-families. Confocal images of stably transfected epimastigotes, shown on Figure 4, demonstrated that, whereas GFP is expressed as a soluble protein present throughout the parasite cytoplasm, (Figure 4A-C) GFP fusions of β1- and δ-amastins are clearly located at the cell surface (Figure 4D-J). Interestingly, a distinct cellular localization, with a punctuated pattern in the parasite cytoplasm of GFP fusion of δ-Ama40 as well as a more disperse distribution within the cytoplasm of the β2- amastin GFP fusion, in addition to their surface localization was observed (Figure 4G-I and M-O) Although all amastin sequences present a N-terminal signal peptide domain, the δ-Ama40 and δ-Ama50 have a C-terminal peptide that is not present in other members of the amastin family (Additional file 2: Figure S2). In spite of these differences, all amastin sequences showed a cellular localization pattern that is consistent with the topology predicted for *Leishmania* amastins as transmembrane proteins [8], as well as with our *in silico* analyses which confirm the presence of four hydrophobic regions, a hallmark for all amastin sequences (Additional file 1: Figure S1B). To further examine their cellular localization, particularly for the δ-Ama40:GFP fusion, which may be associated with intracellular vesicles, we performed co-localization analysis with the glycosomal protein phosphoenolpyruvatecarboxykinase (PEPCK) in immunofluorescence assays. As shown by confocal images presented on Additional file 3: Figure S3, the GFP fusion protein does not co-localize with anti-PEPCK antibodies, indicating that the vesicles containing δ-Ama40 are not associated with glycosomal components. Finally, we also performed immunoblot analyses of sub-cellular fractions of the parasite and compared the presence of GFP-fusions in enriched membrane and soluble fractions of transfected epimastigotes (Figure 5). In agreement with the confocal analyses, the immunoblot results show that all four amastins that were expressed as GFP fusion proteins are presented in membrane enriched fractions.

## Conclusions

Taken together, the results present here provided further information on the amastin sequence diversity, mRNA expression and cellular localization, which may help elucidating the function of this highly regulated family of *T. cruzi* surface proteins. Our analyses showed that the number of members of this gene family is larger than what has been predicted from the analysis of the *T. cruzi* genome and actually includes members of two distinct amastin sub-families. Although most *T. cruzi* amastins have a similar surface localization, as initially described, not all amastins genes have their expression up-regulated in amastigotes: although we confirmed that transcript levels of δ-amastins are up-regulated in amastigotes from different *T. cruzi* strains, β-amastin transcripts are more abundant in epimastigotes than in amastigotes or trypomastigotes. Together with the results showing that, in the G strain, which is known to have lower infection capacity, expression of δ-amastin is down-regulated, the additional data on amastin gene expression presented here indicated that, besides a role in the intracellular, amastigote stage, *T. cruzi* amastins may also serve important functions in the insect stage of this parasite. Hence, based on this more detailed study on *T. cruzi* amastins, we should be able to test several hypotheses regarding their functions using a combination of protein interaction assays and parasite genetic manipulation.

## Methods

### Sequence analyses

Amastin sequences were obtained from the genome databases of *T. cruzi* CL Brener, Esmeraldo and Sylvio X-10 strains [25,26]. The sequences, listed in Additional file 4: Table S1, were named according to the genome annotation of CL Brener or the contig or scaffold ID for the Sylvio X10/1 and. All coding sequences were translated and aligned using ClustalW [27]. Amino acid sequences from CL Brener, Esmeraldo, Sylvio X-10, and *Crithidia* sp (ATCC 30255) were subjected to maximum-likelihood tree building using the SeaView version 4.4 [28] and the phylogenetic tree was built using an α-amastin from *Crithidia* sp as root. Weblogo 3.2 was used to display the levels of sequence conservation throughout the protein [29]. Amino acid sequences from one amastin from each sub-family were used to predict trans membrane domains, using SOSUI [30] as well as signal peptide, using SignalP 3.0 [31]. For copy number estimations, individual reads from the genome sequence of *T. cruzi* CL Brener [13] were aligned by reciprocal BLAST against each amastin coding sequences. Unique reads showing at least 99.7% of identity were mapped on the CDS and the coverage for each nucleotide was determined. Coverage values were normalized through z-score and the copy numbers were determined after determining the ratios between z-score and the whole genome coverage.

### Parasite culture

*T. cruzi* strains or clones, obtained from different sources, were classified according to the nomenclature and genotyping protocols described by [32]. Epimastigote forms of *T. cruzi* strains or clones Colombiana, G, Sylvio X-10, Dm28c, Y and CL Brener were maintained at 28°C in liver infusion tryptose (LIT) medium supplemented with 10% fetal calf serum (FCS) as previously described [3]. Tissue culture derived trypomastigotes and amastigotes were obtained after infection of LLC-MK2 or L6 cells with metacyclic trypomastigotes generated in LIT medium as previously described [3].

### Pulse-field gel electrophoresis and Southern blot analyses

Genomic DNA, extracted from 10^7^epimastigotes and included in agarose blocks were separated as chromosomal bands by pulse-field gel electrophoresis (PFGE) using the Gene Navigator System (Pharmacia) as described by Cano *et al.* (1995) [33], with the following modifications: separation was done in 0.8% agarose gels using a program with 5 phases of homogeneous pulses (north/south, east/west) with interpolation for 135 h at 83 V. Phase 1 had pulse time of 90 s (run time 30 h); phase 2 120 s (30 h); phase 3200 s (24 h); phase 4 350 s (25 h); phase 5 800 s (26 h). Chromosomes from *Saccharomyces cerevisiae* (Bio-Rad) were used as molecular mass standards. Separated chromosomes were transferred to nylon filters and hybridized with ^32^P labelled probes prepared as described in the following section.

### RNA purification and Northern blot assays

Total RNA was isolated from approximately 5 × 10^8^ epimastigote, trypomastigote and amastigote forms using the RNeasy® kit (Qiagen) following manufacturer’s recommendations. RNA samples (15 μg/lane) were separated by denaturing agarose gel electrophoresis, transferred to Hybond-N+ membranes and hybridized with the ^32^P labeled fragments corresponding to each *T. cruzi* amastin sequence as described [3]. The probes used were PCR amplified fragments from total genomic DNA extracted from the CL Brener strain using primers described in Table 1, in addition to a PCR fragment generated by amplification of the insert cloned in plasmid TcA21 (corresponding to δ-amastin) and the 24Sα ribosomal RNA[6]. DNA fragments were labeled using the Megaprime DNA-labeling kit (GE HealthCare) according to the manufacturer’s protocol. All membranes were hybridized in a 50% formamide buffer for 18 h at 42°C and washed twice with 2X SSC/0.1% SDS at 42°C for 30 min each, as previously described [3]. The membranes were exposed to X-ray films (Kodak) or revealed using the STORM840 PhosphoImager (GE HealthCare).

**Table 1:** Sequence of primers used to amplify amastin isoforms ORFs.

**Primer name / gene ID**	**Primer Sequence (5’-3’)**	**Restriction enzyme**
pδ1-amastin (F) Tc00.1047053511071.40	TTGTTCTAGAGTAGGAAGCAATG	*XbaI*
pδ1-amastin (R) Tc00.1047053511071.40	CGCTGGATCCGAACCACGTGCA	*BamHI*
**β1-amastin (F) Tc00.1047053509965.390**	CCTAGGAGGATGTCGAAGAAGAAG	*AvrII*
**β1-amastin (R) Tc00.1047053509965.390**	AGATCTCGAGCACAATGAGGCCCAG	*BglII*
**β2-amastin (F) Tc00.1047053509965.394**	TCTAGATGGGCTTCGAAACGCTTGC	*XbaI*
**β2-amastin (R) Tc00.1047053509965.394**	GGATCCCCAGTGCCAGCAAGAAGACTG	*BamHI*

The underlined sequences correspond to the restriction sites recognized by the restriction enzyme.

### Plasmid constructions

To express different amastin genes in fusion with GFP we initially constructed a plasmid named pTREXAmastinGFP. The coding sequence of the TcA21 cDNA clone [3] (accession number U04339) was PCR-amplified using a forward primer (5’-CATCTAGAAAGCAATGAGCAAAC-3’) and a reverse primer (5’-CTGGATCCCTAGCATACGCAGAAGCAC-3’) containing the *Xba*I and *Bam*HI restriction sites (underlined in the primers), respectively. After digesting the PCR product with *Xba*I and *Bam*HI, the fragment was ligated with the vector fragment of pTREX-GFP [24] that was previously cleaved with *Bam*HI and *Xho*I. To generate the GFP constructions with other amastin genes, their corresponding ORFs were PCR-amplified using the primers listed in Table 1 and total genomic DNA that was purified from epimastigote cultures of *T. cruzi* CL Brener according to previously described protocols [3]. The PCR products were cloned initially into pTZ (Qiagen) and the amastin sequences, digested with the indicated enzymes, were purified from agarose gels with Illustra GFX PCR DNA and Gel Band Purification Kit (GE Healthcare). The fragment corresponding to the TcA21 amastin cDNA was removed from pTREXAmastinGFP after digestion with *Xba*I*/Bam*HI and the fragments corresponding to the other amastin sequences were ligated in the same vector, generating pTREXAma40GFP, pTREXAma390GFPand pTREXAma394GFP. All plasmids were purified using QIAGEN plasmid purification kits and sequenced to confirm that the amastin sequences were properly inserted, in frame with the GFP sequence.

### Parasite transfections and fluorescence microscopy analyses

Epimastigotes of *T. cruzi* CL Brener, growing to a density of 1 to 2 × 10^7^ parasites/mL, were transfected as described by DaRocha *et al.*, 2004 [24]. After electroporation, cells were recovered in 5 ml LIT plus 10% FCS 28°C for 24 h and analysed by confocal microscopy using the ConfocalRadiance2100 (BioRad) system with a 63/100x NA 1.4 oil immersion objective. To perform co-localization analyses, transfected parasites expressing amastin-GFP fusions were prepared for immunofluorescence assays by fixing the cells for 20 minutes in 4% PFA-PBS at room temperature. Parasites adhered to poly-L-lysine coverslips (Sigma) were permeabilized with 0.1% Triton X-100-PBS for 2 minutes, blocked with 4% BSA-PBS for 1 hour and incubated with primary antibodies (rabbit polyclonal antibody anti-phosphoenolpyruvate carboxykinase (anti-PEPCK, kindly provided by Stenio Fragoso, Instituto Carlos Chagas, Curitiba, Brazil) in blocking solution (5.0% non-fat dry milk) for 1 hour followed by incubation with secondary anti-rabbit IgG conjugated with Alexa546. Samples were also stained with 0.1 μg / mL 4’,6-diamidino-2-phenylindole dihydrochloride (DAPI, from Sigma) at room temperature for 5 min before confocal microscopy.

### Parasite membrane fractionation and western blot analyses

Aproximately 10^9^ epimastigotes growing at a cell density of 2 × 10^7^ parasites/mL were harvest, washed with saline buffer (PBS) and ressuspended in lysis buffer (Hepes 20mM; KCl 10 mM; MgCl_2_ 1,5 mM; sacarose 250 mM; DTT 1 mM; PMSF 0,1 mM). After lysing cells with five cycles of freezing in liquid nitrogen and thawing at 37°C, an aliquot corresponding to total protein (T) extract was collected. Total cell lysate was centrifuged at a low speed (2,000 × g) for 10 min and the supernatant was subjected to ultracentrifugation (100,000 × g) for one hour. The resulting supernatant was collected and analysed as soluble, cytoplasmic fraction (C) whereas the pellet, corresponding to the membrane fraction (M) was ressuspended in lysis buffer. Volumes corresponding to 10 μg of total parasite protein extract (T), cytoplasmic (C) and membrane (M) fractions, mixed with Laemmli’s sample buffer, were loaded onto a 12% SDS–PAGE gel, transferred to Hybond-ECL membranes (GE HealthCare), blocked with 5.0% non-fat dry milk and incubated with anti-GFP antibody (Santa Cruz Biotechnology) or anti-PEPCK antibody, followed by incubation with peroxidase conjugated anti-rabbit IgG and the ECL Plus reagent (GE HealthCare).

## Competing interests

The authors declare that they have no competing interests.

## Authors’ contributions

MMK-M, LL and WDR carried out the molecular genetic studies, microscopy analyses, sequence alignments and phylogenetic analyses. RMCP and PRA participated in molecular genetic studies. RPM-Neto and DCB participated in the sequence and phylogenetic analyses. RAM participated in the microscopy analyses. WDR and SMRT designed and coordinated the study and drafted the manuscript. All authors have read and approved the final manuscript.

## Supplementary Material

Additional file 1 **Comparative sequence analysis of*T. cruzi* amastins.** (Figure S1A) Percentages of amino acid identities among all *T. cruzi* amastin sequences present in the CL Brener and Sylvio X-10 genome databases. (Figure S1B) Conserved amino acid residues and conserved domains among sequences corresponding to all amastin genes present in the *T. cruzi* CL Brener genome are represented using the WebLogo software. The x axis depicts the amino acid position. The taller the letter the lesser the variability at the site. Predicted transmembrane domains are underlined. Click here for file

Additional file 2 **Amino acid sequences of delta- and beta-amastins.** (Figure S2) Predicted amino acid sequences of one representative member of δ-amastin, δ-ama40, β1 and β2-amastins present in the *T. cruzi* CL Brener genome. Click here for file

Additional file 3 **Subcellular localization of δ-Ama40 fused with GFP.** (Additional file 3: Figure S3) Permeabilized, stable transfected CL Brener epimastigotes were incubated with anti-PEPCK antibody and a secondary antibody conjugated to Alexa546. GFP (panels A and D), Alexa 546 (B and E) and merged (C and F) fluorescent images were obtained by confocal microscopy of parasites expressing δ-Ama40GFP as described in Figure 4. (Bar = 10 μm). Click here for file

Additional file 4: Table S1 Amastin sequences presented in Figure 1. Click here for file
