# Supplementary material for: Distinct genomic organization, mRNA expression and cellular localization of members of two amastin sub-families present in Trypanosoma cruzi
Source: BMC Microbiol. 2013 Jan 17;13:10. doi: 10.1186/1471-2180-13-10 (PMC3598723; doi:10.1186/1471-2180-13-10)
Supplement: Additional file 1 — Comparative sequence analysis ofT. cruzi amastins. (Figure S1A) Percentages of amino acid identities among all T. cruzi amastin sequences present in the CL Brener and Sylvio X-10 genome databases. (Figure S1B) Conserved amino acid residues and conserved domains among sequences corresponding to all amastin genes present in the T. cruzi CL Brener genome are represented using the WebLogo software. The x axis depicts the amino acid position. The taller the letter the lesser the variability at the site. Predicted transmembrane domains are underlined. [file 1471-2180-13-10-S1.pdf]

(A)

Strain	Haplotype	Class	ID	CL Brener	CL Brener	CL Brener	CL Brener	CL Brener	CL Brener	CL Brener	CL Brener	CL Brener	CL Brener	CL Brener	CL Brener
			Haplotype	Esm-like	Esm-like	Esm-like	Non-Esm	Non-Esm	Non-Esm	Esm-like	Non-Esm	Esm-like	Non-Esm	Esm-like	Non-Esm
			Class	δ-amastin	δ-amastin	δ-amastin	δ-amastin	δ-amastin	δ-amastin	δ-Ama40	δ-Ama50	β1-amastin	β1-amastin	β2-amastin	β2-amastin
Strain	Haplotype	Class	ID	Tc00.1047053507485.130	Tc00.1047053507485.150	Tc00.1047053507739.120	Tc00.1047053506437.10	Tc00.1047053506437.30	Tc00.1047053509051.20	Tc00.1047053511071.40	Tc00.1047053511903.50	Tc00.1047053509965.390	Tc00.1047053509051.20	Tc00.1047053509965.394	Tc00.1047053511497.19
CL Brener	Esm-like	δ-amastin	Tc00.1047053507485.130												
CL Brener	Esm-like	δ-amastin	Tc00.1047053507485.150	**100**											
CL Brener	Esm-like	δ-amastin	Tc00.1047053507739.120	**86**	**86**										
CL Brener	Non-Esm	δ-amastin	Tc00.1047053506437.10	**85**	**85**	**95**									
CL Brener	Non-Esm	δ-amastin	Tc00.1047053506437.30	**85**	**85**	**99**	**95**								
CL Brener	Non-Esm	δ-amastin	Tc00.1047053509289.10	**85**	**85**	**98**	**95**	**98**							
CL Brener	Esm-like	δ-Ama40	Tc00.1047053511071.40	48	48	49	50	50	50						
CL Brener	Non-Esm	δ-Ama50	Tc00.1047053511903.50	50	50	51	51	51	51	**87**					
CL Brener	Esm-like	β1-amastin	Tc00.1047053509965.390	18	18	22	22	22	22	15	20				
CL Brener	Non-Esm	β1-amastin	Tc00.1047053509051.20	18	18	21	21	21	21	15	21	**97**			
CL Brener	Esm-like	β2-amastin	Tc00.1047053509965.394	35	35	31	32	32	32	27	28	25	25		
CL Brener	Non-Esm	β2-amastin	Tc00.1047053511497.19	32	32	31	32	32	32	21	20	17	18	**93**	

Strain	Haplotype	Class	ID	Sylvio	Sylvio	Sylvio	Sylvio	Esm	Esm	Esm	Esm
			Class	δ-amastin	δ-amastin	β1-amastin	β2-amastin	δ-amastin	δ-amastin	β1-amastin	β2-amastin
Strain	Haplotype	Class	ID	sylviocontig_7286	Sylvio-pDelta	sylviocontig_527	sylviocontig_527	scf7180000307809	scf7180000307960	scf7180000308152	scf7180000308152
CL Brener	Esm-like	δ-amastin	Tc00.1047053507485.130	**83**	51	18	34	**86**	48	18	34
CL Brener	Esm-like	δ-amastin	Tc00.1047053507485.150	**83**	51	18	34	**86**	48	18	34
CL Brener	Esm-like	δ-amastin	Tc00.1047053507739.120	**91**	51	24	31	**85**	48	22	31
CL Brener	Non-Esm	δ-amastin	Tc00.1047053506437.10	**92**	50	24	31	**88**	48	24	31
CL Brener	Non-Esm	δ-amastin	Tc00.1047053506437.30	**90**	52	24	31	**85**	49	22	31
CL Brener	Non-Esm	δ-amastin	Tc00.1047053509289.10	**91**	52	21	31	**85**	49	22	31
CL Brener	Esm-like	δ-Ama40	Tc00.1047053511071.40	49	**86**	15	25	50	**96**	15	27
CL Brener	Non-Esm	δ-Ama50	Tc00.1047053511903.50	50	**90**	21	25	50	**87**	20	27
CL Brener	Esm-like	β1-amastin	Tc00.1047053509965.390	20	19	**95**	26	21	11	**89**	25
CL Brener	Non-Esm	β1-amastin	Tc00.1047053509051.20	20	19	**98**	28	24	11	**97**	25
CL Brener	Esm-like	β2-amastin	Tc00.1047053509965.394	31	22	25	**89**	31	28	26	**99**
CL Brener	Non-Esm	β2-amastin	Tc00.1047053511497.19	30	19	18	**94**	31	22	18	**94**

(B)

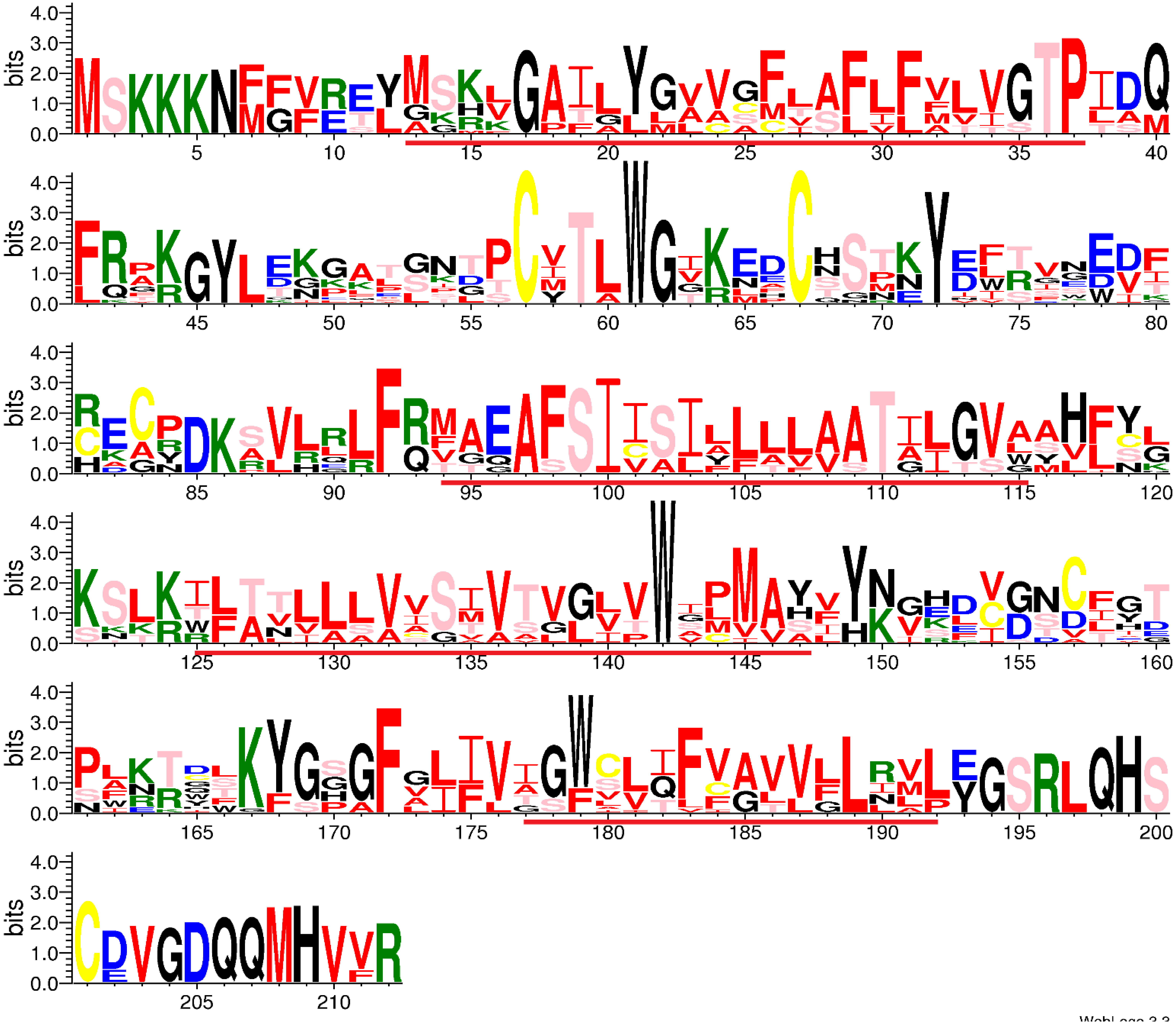

WebLogo 3.3
