# Supplementary material for: Distinct genomic organization, mRNA expression and cellular localization of members of two amastin sub-families present in Trypanosoma cruzi
Source: BMC Microbiol. 2013 Jan 17;13:10. doi: 10.1186/1471-2180-13-10 (PMC3598723; doi:10.1186/1471-2180-13-10)
Supplement: Additional file 2 — Amino acid sequences of delta- and beta-amastins. (Figure S2) Predicted amino acid sequences of one representative member of δ-amastin, δ-ama40, β1 and β2-amastins present in the T. cruzi CL Brener genome. [file 1471-2180-13-10-S2.pdf]

```
δ-amastin    ---------- --MSKLGAIL YGAVGFLAFL FVLVGTPIDQ FRAK---EKG ATGNTPCMTL
δ Ama40      ---------- --MSMVGAIL YGVVSFTALL LMLVGTPLDQ FRFR---TNP LPLIDPCVTL
β1-amastin   MSKKKNFFVR EYGKHKGATG LLLACMVSFI FFVIGTPIAM LQPK------ -LSSGTCYTL
β2-amastin   ------MGFE TLAGRVGPFA YMVCACISFV FATVSTPTSQ FRGKGYLDGG KESKLSCVTA

δ-amastin    WGIKEDCHST KYEFTVGEDF RECP--SVLR LFRMAEAFSI ISILLLLAAT ALGVAAHFCL
δ-Ama40      WGGKLFCSGT EYIISGEDVI HECY--AVLG LFRVAEASSI VSIALLLVAT ILGVLSHLYK
β1-amastin   WGTR-PCNSP NYNWRVNWDT CKAR----RL RFQFGEAFSI CALYFAVVA- GIGSWYVLSG
β2-amastin   WGVKNECTSN KYDLRSSEIK CEGNDKRLHQ LFQTTQAFSI ISIFLTFAS- IITVGMLFNG

δ-amastin    KSLKIFATLL LVVSIVTVGL VWIPMAYFYN -HDVDNCLGT PLKTCSKYGG GFVIIVIGWC
δ-Ama40      KSLRTLTNVL LVSGMVTVGV VWGLMVHVYK -EKIGDCIVT PFKTQLKFGH GFGLIVASWS
β1-amastin   SNKKWLTVLA SAISTVSGLV TWSMVASIHN VKLCGSDTYT SAN--TKYGP GFALFVTGFV
β2-amastin   KSTRRLTLLL AVASVAALLI PWACMAAVYK GSFCGTVFHD NWRRDWKYSS AFGLFLGGWL

δ-amastin    LIFVAVVLLR ML-------- ---------- --
δ-ama40      LTFFAVVFLN LPYGSRLQHS CDVGDQQMHV VR
β1-amastin   VQFCGLLGLI VLE------- ---------- --
β2-amastin   VQLVGTVFLL AL-------- ---------- --
```
