# Supplementary figures and images for: Distinct genomic organization, mRNA expression and cellular localization of members of two amastin sub-families present in Trypanosoma cruzi
Source: BMC Microbiol. 2013 Jan 17;13:10. doi: 10.1186/1471-2180-13-10 (PMC3598723; doi:10.1186/1471-2180-13-10)

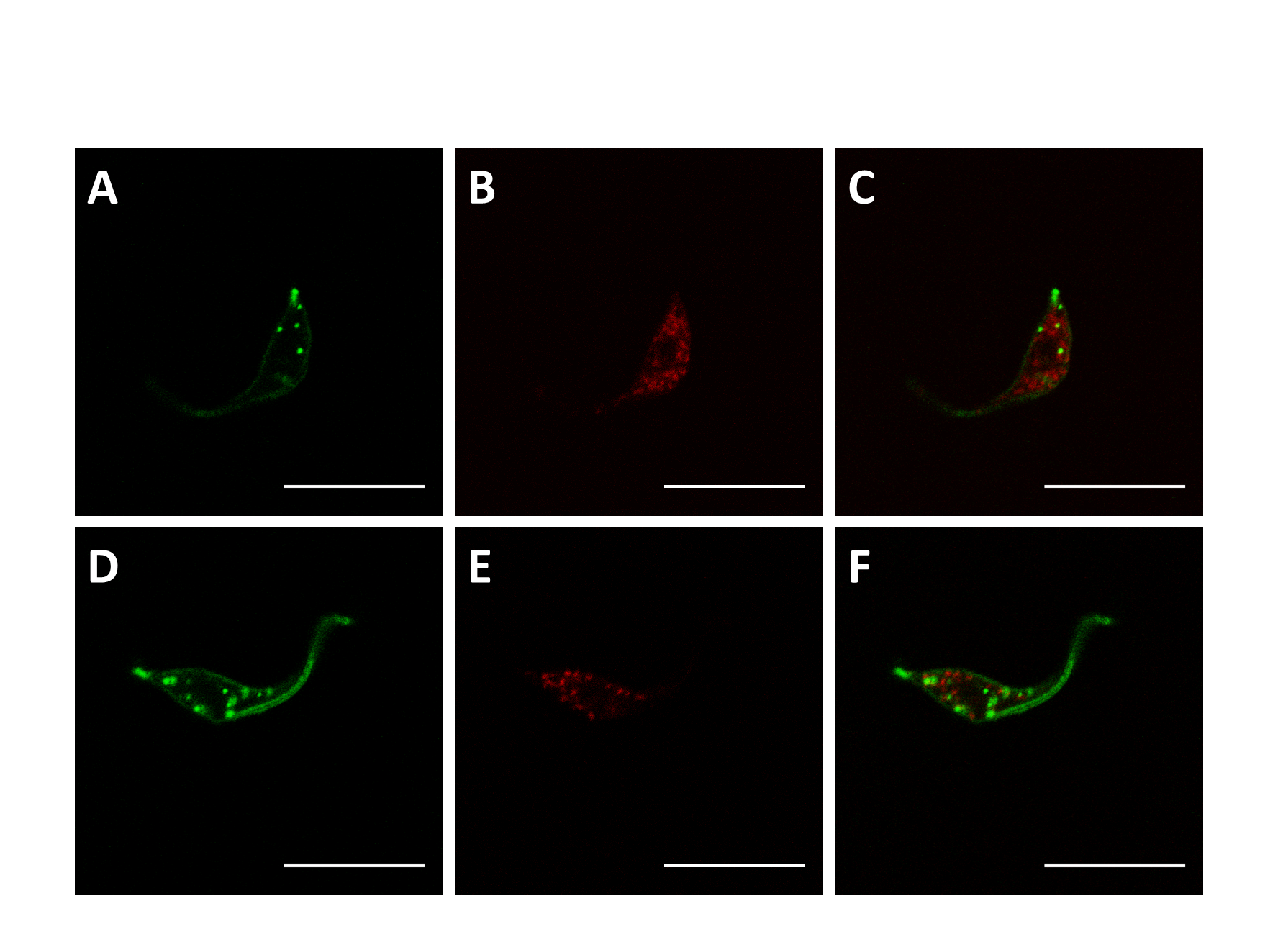

Supplement: Additional file 3 — Subcellular localization of δ-Ama40 fused with GFP. (Additional file 3: Figure S3) Permeabilized, stable transfected CL Brener epimastigotes were incubated with anti-PEPCK antibody and a secondary antibody conjugated to Alexa546. GFP (panels A and D), Alexa 546 (B and E) and merged (C and F) fluorescent images were obtained by confocal microscopy of parasites expressing δ-Ama40GFP as described in Figure 4. (Bar = 10 μm). [file 1471-2180-13-10-S3.tiff]
