# Supplementary material for: Distinct genomic organization, mRNA expression and cellular localization of members of two amastin sub-families present in Trypanosoma cruzi
Source: BMC Microbiol. 2013 Jan 17;13:10. doi: 10.1186/1471-2180-13-10 (PMC3598723; doi:10.1186/1471-2180-13-10)
Supplement: Additional file 4: Table S1 — Amastin sequences presented in Figure 1. [file 1471-2180-13-10-S4.pdf]

Supplementary table 1. **A**mastin sequences used to generate the phylogenetic tree shown in figure 1.

Sequence ID	T. cruzi strain	*Genomic location*	Classification
Tc00.1047053509965.390	CL Brener Esmeraldo-like	*In silico* chromosome - TcChr32-S	β1-amastin
Tc00.1047053509051.20	CL Brener Non-Esmeraldo-like	*In silico* chromosome - TcChr32-P	β1-amastin
Tc00.1047053509965.394	CL Brener Esmeraldo-like	*In silico* chromosome - TcChr32-S	β2-amastin
Tc00.1047053511497.19	CL Brener Non-Esmeraldo-like	*In silico* chromosome - TcChr32-P	β2-amastin
Tc00.1047053507739.120	CL Brener Esmeraldo-like	*In silico* chromosome - TcChr34-S	δ-amastin
Tc00.1047053506437.10	CL Brener Non-Esmeraldo-like	*In silico* chromosome - TcChr34-P	δ-amastin
Tc00.1047053507485.150	CL Brener Esmeraldo-like	*In silico* chromosome - TcChr34-S	δ-amastin
Tc00.1047053506437.30	CL Brener Non-Esmeraldo-like	*In silico* chromosome - TcChr34-P	δ-amastin
Tc00.1047053507485.130	CL Brener Esmeraldo-like	*In silico* chromosome - TcChr34-S	δ-amastin
Tc00.1047053509289.10	CL Brener Non-Esmeraldo-like	*In silico* chromosome - TcChr34-P	δ-amastin
Tc00.1047053511071.40	CL Brener Esmeraldo-like	*In silico* chromosome - TcChr26-S	δ-ama40/50
Tc00.1047053511903.50	CL Brener Non-Esmeraldo-like	*In silico* chromosome - TcChr26-P	δ-ama40/50
Esm_scf7180000308152b	Esmeraldo	Scaffold - scf7180000308152 (Length = 144,278 bp)	β1-amastin
Esm_scf7180000308152a	Esmeraldo	Scaffold - scf7180000308152 (Length = 144,278 bp)	β2-amastin
Esm_scf7180000307809	Esmeraldo	Scaffold - scf7180000307809 (Length = 31,745 bp)	δ-amastin
Esm_scf7180000307960	Esmeraldo	Scaffold - scf7180000307960 (Length = 49,875 bp)	δ-ama40/50
Sylviocontig_527b	Sylvio X10/1	Contig - sylviocontig_527 (Length = 9,484 bp)	β1-amastin
Sylviocontig_527a	Sylvio X10/1	Contig - sylviocontig_527 (Length = 9,484 bp)	β2-amastin
Sylviocontig_7286	Sylvio X10/1	Contig - sylviocontig_7286 (Length = 959 bp)	δ-amastin
Sylvio_IR	Sylvio X10/1	Assembling of individual reads (this work)	δ-ama40/50
